# Pamphlets Received

**Published:** 1889-07

**Authors:** 


PAMPHLETS RECIEVED.—American Public Health Association Announcement of the Seventh annual meeting, to be held at Brooklyn, N. Y., Oct. 22, 1889.

